# Metrics of Phenotypic Aging From the Energetics Perspective

**DOI:** 10.1093/geroni/igaa057.469

**Published:** 2020-12-16

**Authors:** Pei-Lun Kuo, Michelle Shardell, Jennifer Schrack, Morgan Levine, Eleanor Simonsick, Luigi Ferrucci

**Affiliations:** 1 National Institute on Aging, Bethesda, Maryland, United States; 2 University of Maryland School of Medicine, Baltimore, Maryland, United States; 3 Johns Hopkins Bloomberg School of Public Health, Baltimore, Maryland, United States; 4 Yale University School of Medicine, New Haven, Connecticut, United States

## Abstract

Identifying the most critical metrics of aging is an ongoing challenge due to a lack of comprehensive measurements and heterogeneity of the aging process. Using the Baltimore Longitudinal Study of Aging, we developed a conceptual framework to identify metrics of aging that capture the hierarchical and temporal relationships between functional aging, phenotypic aging, and biological aging based on four hypothesized domains: energy regulation, body composition, homeostatic mechanisms, and neurodegeneration. Focusing on the energetics domain, we examined trajectories of eight phenotypes using more than 10 years of longitudinal data. The standardized Cronbach’s alpha for these variables was 0.80, providing construct validity of our concept. We further implemented item response theory to integrate these phenotypes into a summarized energy score. Linear mixed models were used to assess the cross-sectional and longitudinal associations between the summarized energy score and physical functioning as measured by gait speed and time to walk 400m as quickly as possible (number of participants ~ 811, number of observations ~ 1700). After adjusting for age, sex, weight, and height, a higher summarized energy score was independently associated with faster baseline gait speed (0.13 m/s, p<0.001 ) and faster 400m time (-35.3 seconds, p<0.001), and longitudinally associated with slower gait speed decline (0.08 m/s/decade, p<0.001) and slower 400m time increase (-37.8 secs/decade, p<0.001). This work demonstrates the utility of our energetics domain-based summarized score. Moving forward, it will be important to clarify relationships between this summarized score and other functional metrics and assess its generalizability to the other cohorts.

